# Adaptive Door Opening Control Algorithm of Bio-Inspired Mobile Manipulator Based on Synchronous Sensing

**DOI:** 10.3389/fbioe.2021.821981

**Published:** 2022-01-21

**Authors:** Wenping Wu, Wansu Liu

**Affiliations:** Information Engineering Department, Suzhou University, Suzhou, China

**Keywords:** prediction model, bio-inspired mobile manipulator, synchronous sensing, adaptive door opening control, sliding mode control

## Abstract

At present, the research of robot door opening method is basically realized by identifying the door handle through the synchronous sensing system on the premise that the bio-inspired mobile manipulator is located in front of the door. An adaptive door opening strategy of a bio-inspired mobile manipulator based on a synchronous sensing system is proposed. Firstly, the random delay distribution in clock synchronization technology is analyzed in detail, and its distribution is verified on the experimental platform of adjacent nodes. Based on the Gaussian distribution of random delay, the relative frequency offset and relative phase offset of adjacent nodes are calculated. The clock synchronization of network cable sensor nodes is realized. Secondly, based on the information data of synchronous sensing system, this article realizes target detection and tracking based on depth network. In addition, based on the sliding mode control theory, the dynamic model of the nonholonomic bio-inspired mobile manipulator is applied. Finally, a robust adaptive sliding mode control method for nonlinear systems with input gain uncertainty and unmatched uncertainty is proposed by combining adaptive backstepping with sliding mode control. By adding sliding mode control in the last step of adaptive backstepping, the uncertainty of the system is compensated, and the system trajectory is maintained on the specified sliding mode manifold. The tracking control and stability control of the nonholonomic bio-inspired mobile manipulator are simulated. The experimental and simulation results show that the control method proposed in this article is effective and feasible.

## 1 Introduction

In recent years, with the rapid development of robot technology, robots are playing a more and more important role in people’s life. All kinds of robots have been widely used in national defense, industry, life, medicine, and other fields (Ahmad and Abbas 2020). Many high-tech automatic production lines use mechanical arms to help with loading, assembly, and other production operations (Almesaeed et al., 2021). For example, the automotive industry also uses mechanical arms to replace people in car body spraying, welding, and other work. Although robots can help us do a lot of repetitive work, if they are used to deal with more complex work, such as disaster relief and handling of dangerous goods, as well as dangerous work such as deep-sea exploration, it needs to involve quite complex image recognition and robot positioning (Ding et al., 2019; Lundeen et al., 2019). Most of them are operated manually rather than by robots. As a complex and practical robot, a manipulator results from the intersection and integration of various disciplines (Habibzadeh et al., 2018). In the traditional sense, the control mode of the robot is basically realized by manual control with a remote controller and operating handle, and its flexibility is poor (Brahmi et al., 2021). In this study, the natural body sensing control technology is introduced into the control of the manipulator and the control mode based on synchronous sensing technology (Tolosana et al., 2020). This non-contact adaptive control of the manipulator has great practical value. To a certain extent, the innovation of robot control mode is realized, making the robot control more diverse and flexible. Therefore, the research on this subject has certain engineering practical significance (Lu et al., 2020).

For the manipulator adaptive control of synchronous sensing system, the design of synchronous sensing system is also very important. Scholars have designed various corresponding time synchronization algorithms to solve problems (Jiang et al., 2021a; Xiao et al., 2021). Reference broadcast synchronization (RBS) is designed by Jiang et al. (2018), a receiver synchronization protocol. In the RBS protocol, the reference node sends broadcast information to adjacent nodes. After receiving the broadcast information, these adjacent nodes exchange their own timestamp information, and then the parameters required by the nodes are estimated by the linear regression method. Liu et al. (2020) designed a timing synch protocol for sensor networks (TPSN) protocol, a sender-receiver protocol. In this protocol, wireless sensor networks are dispersed in the form of a tree. The clock synchronization of the whole network is completed by synchronizing each node with its upper parent node. The mutual communication between each node and its parent node is realized by a two-way information exchange to compensate for the clock deviation. The flooding time synchronization protocol (FTSP) proposed by Nakanishi et al. (2020) assigns an ID number to each sensor node, completes the clock synchronization between the sending node and the receiving node through broadcasting synchronization packets, realizes the clock synchronization of the whole network by hierarchical classification, and uses linear regression deviation compensation to compensate for the relevant error source. The time diffusion protocol (TDP) proposed by Outón et al. (2019) is based on the iterative weighted average method and uses the information diffused by the sensor nodes involved in the whole network in the synchronization process. However, asynchronous diffusion protocol (ADP) uses the same diffusion method as TDP. However, the protocol in the sensor node is asynchronously completed when executed, and it will be independently completed when correcting the time of the node. Mo et al. (2020) proposed pairwise broadcast synchronization (PBS), a new information exchange mechanism. PBS uses two synchronization methods, sender-receiver and receiver only, to achieve energy-efficient network-wide synchronization. In this protocol, the common sensor node uses the timestamp information of the super node to synchronize the local clock (Zhao et al., 2021). The synchronization method reduces the total energy consumption of the whole network by reducing the times of exchanging timestamp information between nodes. Since less information is required to realize clock synchronization, it significantly reduces the energy consumption compared with RBS, tpsn, and other protocols. The advantage of paired broadcast synchronization protocol is particularly obvious when sensor nodes are densely deployed. Asfour et al. (2019) designed a distributed consistent clock synchronization algorithm (CCS). The algorithm aims to reduce the clock error between geographically adjacent nodes and keep all nodes at a common tilt rate to achieve long-term clock synchronization. It completes the parameter estimation of paired nodes by one-way broadcasting and then weights and averages the parameter estimation to realize the correction of the local clock.

The manipulator control system is a complex control system, which integrates many disciplines, such as computer, machinery, control, and electrical disciplines (Bostelman et al., 2018). In recent years, with the development of synchronous sensing technology, it has been gradually applied in the field of scientific research. Now, many researchers and engineers propose to apply body-sensing technology to the control of the robot to realize the research and design of a natural and non-contact human–computer interaction control system. Chen et al. (2021a) determined the position and direction of the door handle based on Bayesian a posteriori estimation based on force measurement. Assuming that the handle does not need to twist and the 3D model of the handle is known, Wu et al. (2020) designed a static manipulator to open the cabinet door and drawer. Liu et al. (2021a) ensured that the manipulator could perform the opening action by setting a series of behaviour modes after prepositioning the door handle range (20 cm). Furthermore, the tracking control of the bio-inspired mobile manipulator has been a hot issue in recent years. There are many control strategies to solve this problem. When the dynamics of the system is known, the commonly used control strategies include nonlinear feedback control, input-output feedback linearization, and computational torque control. These methods need to know the dynamic model of the system and ignore the dynamic uncertainty and external interference of the system. In order to solve this problem, many scholars have also done relevant work. Prianto et al. (2020) designed a robust controller based on Lyapunov stability theory for the case of system dynamic coupling and system inertia parameter uncertainty. Liu et al. (2021b) proposed neural network adaptive control, in which a neural network is used to estimate the dynamic coupling and parameter uncertainty of the system. When the dynamic parameters of the system are completely unknown, a robust damping controller is designed by Chen et al. (2021b). The algorithm can suppress the bounded disturbance in the system. Sliding mode control has strong robustness to the uncertainties and external disturbances of the system, so it is very suitable for the tracking control of the bio-inspired mobile manipulator. de Gea Fernández et al. (2017) and Goswami et al. (2021) proposed a sliding mode controller based on a neural network for the tracking control of an omnidirectional bio-inspired mobile manipulator. Hao et al. (2021) proposed a sliding mode control based on backstepping. The controller can quickly make the tracking error close to zero, make the tracking process more stable, and reduce the chattering of the system (Yang et al., 2021).

In order to overcome the above problems, the main contributions of this article are summarized as follows: 1) based on the Gaussian distribution of random delay, the relative frequency offset and relative phase offset of adjacent nodes are calculated to realize the clock synchronization of the network cable sensor nodes; 2) the location of target gate is detected and tracked by using synchronous sensor network and deep learning; 3) combining adaptive backstepping with sliding mode control, a robust adaptive sliding mode control method for nonlinear systems with input gain uncertainty and mismatch uncertainty is proposed to control the bio-inspired manipulator.

In this article, we construct a wireless sensor network with clock synchronization. Based on the random delay distribution obeying the Gaussian distribution, the relative frequency offset and relative phase offset of adjacent nodes are calculated to achieve the effect of clock synchronization. In addition, for the trajectory tracking control of the nonholonomic bio-inspired mobile manipulator, an adaptive sliding mode control strategy with an online estimation of system parameters is proposed. In this method, the bio-inspired mobile manipulator is considered a complete system. Firstly, the sliding mode controller is designed according to the dynamic equation of the system; then, an adaptive law is designed to estimate the uncertain parameters in the system. Compared with traditional sliding mode control, the algorithm does not need to know the upper bound of system parameters. Finally, a simulation example shows the effectiveness and superiority of the proposed method.

## 2 Architecture Design of Bio-Inspired Mobile Manipulator Based on Synchronous Sensing

### 2.1 Architecture Design of Bio-Inspired Mobile Manipulator Based on Synchronous Sensing

**FIGURE 1:**
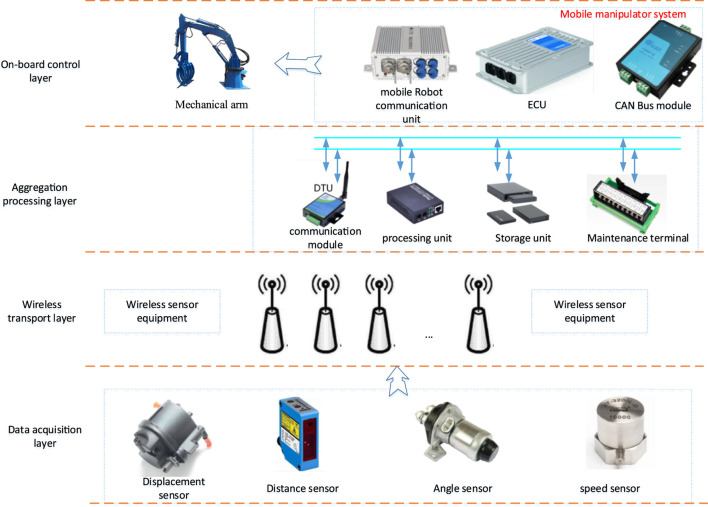
The structure design of bio-inspired mobile manipulator based on synchronous sensing.

Wireless sensor network is a network system composed of a large number of wireless sensor nodes deployed in the monitoring area and formed by wireless communication (Xia et al., 2018; Wu et al., 2019). Its purpose is to collaboratively perceive, collect, and process the object information in the network coverage and then send it to the monitor. Therefore, the design of a single network wireless sensor node cannot be separated from the constraints of the whole network environment. Wireless sensor nodes are a kind of miniaturized computer system. For wireless sensor networks, the number is huge, and batteries are usually used to provide energy. After the energy of sensor nodes is exhausted, they cannot carry out data acquisition, which will directly affect the robustness and life cycle of the whole wireless sensor network. Therefore, the wireless sensor node is an important research aspect in wireless sensor networks. Therefore, the door opening adaptive control system of bio-inspired mobile manipulator based on synchronous sensing designed in this article is shown in Figure 1.

The architecture mainly includes data acquisition layer, wireless transmission layer, aggregation processing layer, and vehicle control layer. The aggregation processing layer and the vehicle control layer are the most important links, in which the aggregation processing layer is mainly used for data fusion, complementing and eliminating redundant information in time and space, and recombining according to the set conditions. Generally, data fusion helps increase the reliability and effectiveness of data, save channel bandwidth, and prolong the network life cycle. In addition, the onboard control layer mainly uses information for adaptive door closing control.

The application background requires the selection, accuracy, and acquisition frequency of sensors, as well as the protocol, frequency band, and transmission distance used in wireless communication. The power technology restricts the energy consumption of sensor technology and wireless communication technology. At the same time, it also requires the power consumption of the processor itself. Sleep technology is essential to the selection and application of sensors. The acquisition frequency required by the application background and the protocol of wireless communication technology put forward specific requirements for the processor’s processing capacity, data acquisition speed and accuracy, and on-chip resources. Therefore, as the core component of the wireless sensor node, the selection of the processor is restricted by all technical requirements.

### 2.2 Research on Clock Synchronization Technology in Sensor Networks

Each sensor node has its own local clock. By detecting the zero-crossing intersection of the periodic output signal of the local oscillator, the sensor node increases the count value in its counter. When the count value reaches a specified value, the interrupt response generates a clock signal, and then the counter will refill the initial value and start a new round of counting. Therefore, the physical clock model is expressed as follows:

$$c\left(t\right)=\lambda \int_{t_{0}}^{t}\omega \left(\tau \right)dt+c\left(t_{0}\right),$$ (1)

where t is the standard time, c(t) is the clock time maintained by the crystal oscillator at time *t*; $c\left(t_{0}\right)$ is the initial clock is the node clock at $t_{0}$ ; ω(τ) is the true frequency of the crystal oscillator; λ are parameters related to the characteristics of the crystal oscillator.

**FIGURE 2:**
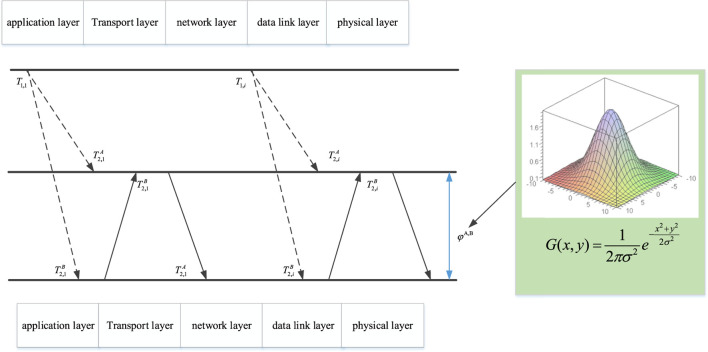
RRS model considering the Gaussian distribution delay.

In this article, the receiver-receiver synchronization method is used to construct the synchronous sensor network. Consider a reference node R and any nodes A and B within the communication range of the reference node. At time *i*, node R sends the *i*th timestamp information to node A and node B, then node A and node B record the arrival time of the timestamp according to their own schedule, and after that node A and node B exchange timestamp information with each other. The clock model of the synchronization method of the receiver-receiver is shown in Figure 2. When the step message is transmitted in the wireless channel, there will be various delays. However, in practice, there are other delays, including interrupt processing, encoding, and decoding delays. However, these delays can be determined, so we will not consider them when designing the algorithm. Therefore, Gaussian time delay can be used to process.

At time i , the arrival time $T_{2,i}^{A}$ at node A can be expressed as

$$T_{2,i}^{A}=T_{1,i}+d^{RA}+\varepsilon_{x_{i}}^{RA}+\varphi^{RA}+f^{RA}\left(T_{1,i}-T_{1,1}\right),$$, (2)

where $T_{1,i}$ is the time information sent by the reference node; $d^{RA}$ and $\varepsilon_{x_{i}}^{RA}$ are fixed delay and random delay, respectively. Similarly, the arrival time information at node B is

$$T_{2,i}^{B}=T_{1,i}+d^{RB}+\varepsilon_{x_{i}}^{RB}+\varphi^{RB}+f^{RB}\left(T_{1,i}-T_{1,1}\right).$$ (3)

In wireless sensor networks, the two main factors affecting the accuracy of clock synchronization are the correct estimation of phase offset and frequency offset by sensor nodes and the propagation delay of synchronization messages. The propagation delay may be much greater than the clock synchronization accuracy we need. Therefore, it is necessary to study the exact causes and impact of these errors. Figure 2 shows the relationship between the delay factor and the network level. Three quantities are random, namely, transmission time, access time, and reception time. However, the other three quantities are fixed, namely, transmission time, propagation time, and reception time. Set reference node R and node A. Uplink delay and downlink delay are defined as

$$\left\{ \begin{array}{l} U_{i}=T_{2,j}-T_{1,j}\\ V_{i}=T_{4,j}-T_{3,j} \end{array}\right..$$ (4)

In *i*th time information interaction, the uplink and downlink delays are

$$\left\{ \begin{array}{l} U_{i}=\left(f-1\right)T_{1,j}+{\dot{\varepsilon }}_{x}+\varphi_{t}\\ V_{i}=\left(f-1\right)T_{3,j}+{\dot{\varepsilon }}_{y}-\varphi_{t} \end{array}\right.,$$ (5)

where ${\dot{\varepsilon }}_{x}$ and ${\dot{\varepsilon }}_{y}$ have accordance with Gaussian distribution and $\varphi_{t}$ is the clock phase offset close to 0.

### 2.3 Research on Target Recognition Algorithm Based on Synchronous Sensor Network

**FIGURE 3:**
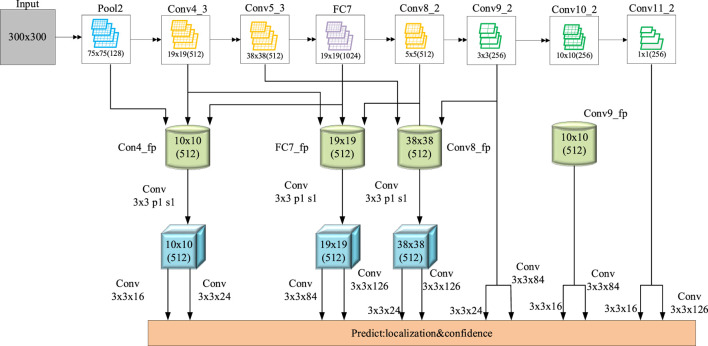
Design of target detection network based on synchronous sensor network.

Compared with traditional two-dimensional image information, depth image information has no special restrictions on the physical and geometric features of external objects or environment, such as lighting conditions (Jiang et al., 2021b). The distance information of the object or environment can be directly obtained using the obtained depth information, which greatly reduces the difficulty of recognition, positioning, and navigation (Sun et al., 2019). The door opening judgment method based on depth image information proposed in this article includes six parts: depth image acquisition, registration, position selection, preprocessing, fitting plane, and door opening judgment. The main content of the research is to judge the door opening based on the depth image, so it will be very important to select the position of the depth image. Whether the position can correctly represent the current state of the door will directly affect the final judgment result. The size of the door is length (2.03 m) × width (0.9 m), the door handle is located on the left of the door body, and the installation height is 0.92 M. Therefore, selecting the upper right of the door handle position will be able to more accurately describe the state information of the door. The most important thing is to build a target recognition network based on the depth image. The depth network structure designed in this article is shown in Figure 3.

For a 3 × 3, when *d* = 1, the corresponding convolution kernel size is 3. When *d* = 24, the corresponding convolution kernel size is 49 (Xu et al., 2020). The expansion rates are {1, 6, 12, 18, 24}, respectively, forming a cavity convolution group with gradient parallel structure, as shown in Figure 3. By setting different expansion rates, the size of the receptive field is expanded dozens of times on the original basis, and the characteristic images of the corresponding connection stage will respectively pass through the hole convolution in this hole convolution group in parallel to obtain the huge target in the SAR image. In addition, a feature transfer layer is formed between each two stages, and a hole convolution group is added between the last two stages to form a feature transfer network, which is conducive to the network to extract the cross-scale target features of SAR image. Then, in the top-down network, the feature map with stronger high-level semantic information is upsampled to generate a feature map with higher resolution. Finally, the feature maps with the same space size in the bottom-up and top-down processes are fused through horizontal connection, mainly using 1 × 1, which can make full use of the underlying positioning details. The low-resolution feature map is upsampled twice, and the upsampling map is combined with the corresponding bottom-up map to reduce the aliasing effect.

## 3 Tracking Control of Bio-Inspired Mobile Manipulator Based on Adaptive Sliding Mode

### 3.1 The Dynamic Model of Bio-Inspired Mobile Manipulator

**FIGURE 4:**
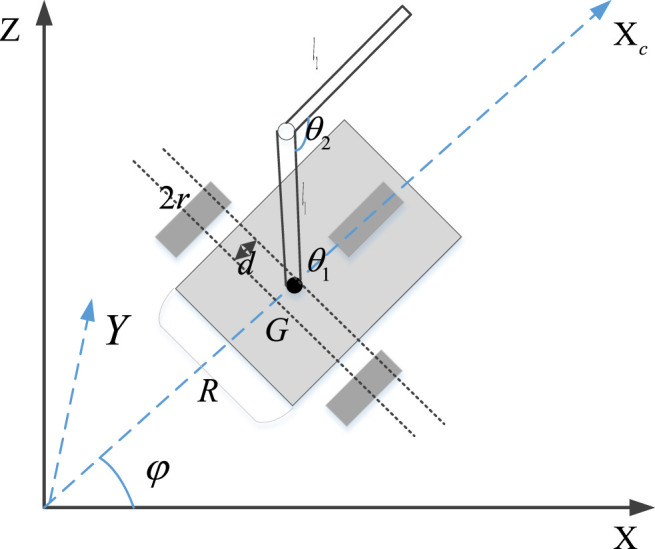
The model diagram of a two-link bio-inspired mobile manipulator.

The mathematical model of bio-inspired mobile manipulator includes kinematic model and dynamic model. The purpose of establishing the dynamic model of the robot system is multifaceted. The forward problem of dynamics is related to the simulation and research of the robot system. The inverse problem is to realize the optimal control using the dynamic model to achieve good dynamic performance and optimal index. The application object of this research is the wheeled bio-inspired mobile manipulator system with nonholonomic constraints, as shown in Figure 4.

Consider the two-link space bio-inspired mobile manipulator shown in Figure 4, in which independent motors drive the two rear wheels of the mobile platform, respectively. A two-link manipulator is installed at the center of mass C of the mobile platform, and the position of its hinge is driven by a motor, respectively, in which the connecting rod 1 can rotate around the *z*-axis and the connecting rod 2 can rotate up and down. During the movement of the mobile platform, it is constrained by non-integrity (the wheels roll with the ground without sliding), while the manipulator is constrained by integrity. The non-integrity constraints on the mobile platform are

$${\dot{y}}_{c}\cos\varphi -{\dot{x}}_{c}\sin\varphi -d\dot{\varphi }=0,$$ (6)

where $\left(x_{c},y_{c}\right)$ is the coordinate of the platform centroid C in the world coordinate system *o*-XYZ, *D* is the distance between the center point G of the two rear wheels and the platform centroid C, and *φ* is the included angle between the symmetry axis direction of the mobile platform and the *x*-axis.

Select the state vector of the system as $q=\left[x_{c},y_{c},\varphi ,\theta_{1},\theta_{2}\right]$ , where $\theta_{1},\theta_{2}$ represent the rotation angle of connecting rod 1 and connecting rod 2, respectively. Then, Eq. 1 can be expressed as

$$A\dot{q}=0,$$ (7)

where A=[−sin⁡φ,cos⁡φ,−d,0,0] .

The dynamic equation of the system can be obtained by the Lagrange energy method:

$$M\left(q\right)\ddot{q}+C\left(q,\dot{q}\right)\dot{q}+F\left(q,\dot{q}\right)+A^{T}\left(q\right)\lambda +\tau_{d}=B\left(q\right)\tau ,$$ (8)

where $q,\dot{q},\ddot{q}\in R^{5}$ are the state quantities of the bio-inspired mobile manipulator, which represent the position, velocity, and acceleration vectors of the system, respectively; M(q) is the inertia matrix of the system and is a positive definite symmetric matrix; $C\left(q,\dot{q}\right)$ is the matrix representing the centripetal force and Coriolis force; $F\left(q,\dot{q}\right)$ is the term of gravity and friction; $\tau_{d}$ is the bounded external interference; B(q) is the input transformation matrix; $A^{T}\left(q\right)$ is the constraint matrix; and λ is the Lagrange multiplier of the corresponding constraint.

Equation 3 contains the dynamic coupling between the mobile platform and the manipulator. Herein, the bio-inspired mobile manipulator is taken as a whole so that the dynamic coupling of the system can be considered. Then, the torque control law is designed based on the dynamic model of the system. According to the matrix theory, there is a full rank matrix. At this time, the kinematic equation of the system can be expressed as

$$M\left(q\right)\ddot{q}+C\left(q,\dot{q}\right)\dot{q}+F\left(q,\dot{q}\right)+A^{T}\left(q\right)\lambda +\tau_{d}=B\left(q\right)\tau ,$$ (9)

Equation 3 contains the dynamic coupling between the mobile platform and the manipulator. Herein, the bio-inspired mobile manipulator is taken as a whole so that the dynamic coupling of the system can be considered. Then, the torque control law is designed based on the dynamic model of the system. At this time, the kinematic equation of the system can be expressed as

$$\dot{q}=Sv,$$ (10)

where $v=\left[\begin{array}{cccc} {\dot{\theta }}_{L} & {\dot{\theta }}_{R} & {\dot{\theta }}_{1} & {\dot{\theta }}_{2} \end{array}\right]$ . In order to eliminate the binding force in Eq. 3, the unconstrained dynamic equation can be obtained as follows:

$$\bar{M}\ddot{z}+\bar{C}\dot{z}+\bar{F}+{\bar{\tau }}_{d}=\bar{\tau },$$ (11)

### 3.2 Design of Adaptive Sliding Mode Controller

**FIGURE 5:**
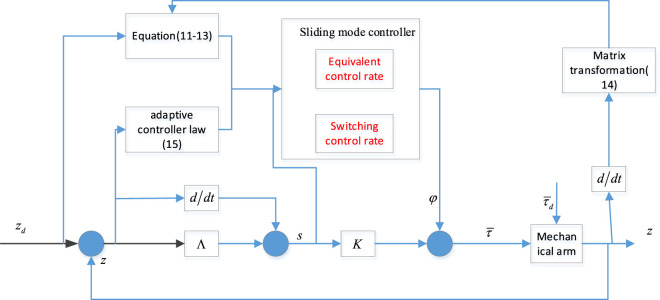
The structure figure of adaptive sliding mode controller.

For the bio-inspired mobile manipulator shown in Figure 5, our control task can be described as follows: given the reference trajectory $z_{d}$ , design the corresponding torque control law so that the actual running trajectory Z of the bio-inspired mobile manipulator can track the given reference trajectory $z_{d}$ . That is, for any defined initial state, $\underset{t\to \infty }{\lim}\left(z_{d}-z\right)=0$ . In this article, the unified control mode is adopted in the design of the controller. In other words, the mobile platform and the manipulator are considered as a whole, and the dynamic coupling of the system is considered in the design of the controller.

Assuming that the desired trajectory of the bio-inspired mobile manipulator is $z_{d}$ , the tracking error of the system is

$$e=z_{d}-e,$$ (12)

The sliding mode function of the system is defined as

$$s=\dot{e}+\Lambda e,$$ (13)

where $\Lambda =\beta I_{4\times 4}$ , β>0 , is the parameters that need to be adjusted in the controller design. The eigenvalues of Eq. 13 are located in the left half-open complex plane, so matrix Λ can ensure that the sliding mode is stable:

$$H\left(q,\dot{q},t\right)=\bar{M}\left(q\right)\left({\ddot{z}}_{d}-\Lambda \dot{e}\right)+\bar{C}\left(q,\dot{q}\right)\left({\dot{z}}_{d}-\Lambda e\right)+\bar{F}\left(q\right)+{\bar{\tau }}_{d},$$ (14)

Assumption 1
$H\left(q,\dot{q},t\right)$ is bounded. There is a bounded function such that for any $H\left(q,\dot{q},t\right)$ & the following conditions are satisfied.

The torque controller of the bio-inspired mobile manipulator system is

$$\left\{ \begin{array}{l} \bar{\tau }=-\text{Ks}-\mu \\ \mu =\frac{s\lambda^{2}\left(q,\dot{q}\right)}{\left\|s\right\|\lambda \left(q,\dot{q}\right)+\varepsilon } \end{array}\right.,$$ (15)

where K is the control parameter of driving torque and *ε* > 0 is the given constant, which is introduced *ε* to reduce the effect of buffeting. For any initial error E (0), the designed controller can ensure that the tracking error *e*(t) is uniformly ultimately bounded (UUB). That is, the stability of the system is guaranteed.

It is required that the upper bound of system parameters in Eq. 12 is determined, but in the actual system, these parameters are uncertain and may change over time. If the method of directly giving these parameters is adopted, although it can also ensure a better tracking effect, this method will degrade the control performance when these parameters change greatly. Adaptive control is to solve the problem of unknown parameter estimation. It uses adaptive law to identify the system parameters online to realize the real-time adjustment of controller parameters. In order to reduce the influence of parameter uncertainty on the controller’s performance, this section gives the design steps of the corresponding adaptive sliding mode controller when the upper bound of the system parameters is unknown. The controller proposed in this article combines the advantages of sliding mode control and adaptive control. It has a strong anti-interference ability and can estimate the uncertain parameters in the system.

Next, an adaptive law is designed to estimate the upper bound in Eq. 11, and the estimated value is $\hat{\Theta }$ . Then the estimated value of the upper bound of the system parameters $\overset{⌢}{\lambda }\left(q,\dot{q}\right)={\hat{\Theta }}^{T}\Lambda$ , parameter $\hat{\Theta }$ . The adaptive law is designed as follows:

$$\phi =\frac{s{\overset{⌢}{\lambda }}^{2}\left(q,\dot{q}\right)}{\left\|s\right\|\overset{⌢}{\lambda }\left(q,\dot{q}\right)+\varepsilon },$$ (16)

## 4 Simulation Results and Performance Analysis

In the simulation test, the time stamp sending cycle is set to 1s, and the test data are 500 observation points. Due to the difference between the start-up time of nodes and the time of establishing connections, we found in many experimental results that the initial phase difference of adjacent nodes is generally between 150 and 250 ms. Therefore, in the simulation experiment, we took the average value and set the initial phase difference to 200 ms to test the performance of the synchronous sensor network.

The manipulator door opening experiment studied in this article mainly includes three parts: first, how to locate the door handle by MT_A, that is, knowing where the door handle is and the distance between the door handle and the body; secondly, after positioning the door handle, combined with its own motion characteristics, how to correctly operate MT_A to the best position in front of the door; and finally, after MT_A reaches the best position in front of the door, according to the structure of the actuator at the end of the manipulator, the forward kinematics is used to plan the rotation component of each joint of the manipulator to enable the manipulator to operate the door handle with correct execution action and complete the door opening experiment.

### 4.1 Clock Verification of Synchronous Sensor Networks

**FIGURE 6:**
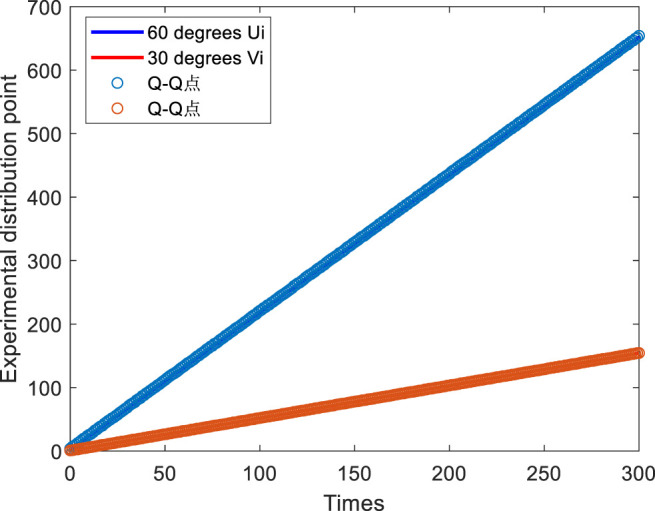
The random delay distribution diagram of the link layer.

For clock verification of synchronous sensor networks according to the above theoretical model of clock synchronization in wireless sensor networks, the actual paired sensor nodes are experimentally verified. Firstly, the basic test working conditions of the two nodes are set. After that, the timestamp information data is actually collected. The experiment set up a coordinator node and a terminal node based on the ZigBee network to establish a paired two-way information exchange mode. Local timestamp information is marked on the timers of the coordinator and the terminal node to exchange the local clocks between them. The coordinator and terminal node are wireless networking based on ZigBee technology, which are implemented in a modular and cascade manner. The random delay distribution of uplink Ui and downlink Vi is analyzed through the collected 10 groups (1,000 times in each group). It is verified that the random delay distribution obeys the normal distribution through the Q-Q diagram. Figure 6 shows that the random delay distribution of uplink Ui and downlink Vi is fitted by Q-Q diagram followed by a normal distribution, and the fitting degree is very high.

As shown in Figure 6, in the experiment, the mean value of uplink and downlink is mainly about 15 because when we collect data, we designate the channel as ZigBee channel ch25, and there is no channel interference (such as signal preemption, signal waiting, signal frequency hopping), and the random delay part is much smaller than the fixed delay part. Therefore, this 20 ms is mainly consumed in the fixed delay, and the mean value of the random delay distribution tends to zero.

**FIGURE 7:**
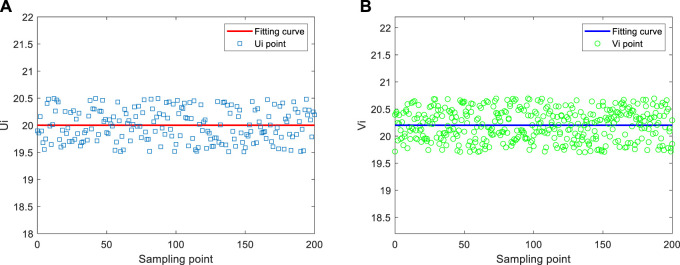
The random delay distribution diagram of the link layer. **(A)** Ui data fitting. **(B)** Vi data fitting.

The time difference between uplink and downlink is used to prove the effectiveness of clock synchronization after compensating clock frequency offset and clock phase offset. After executing the clock synchronization algorithm based on the two-way information exchange model, 300 groups of timestamp information are sampled again. The results are shown in Figure 7.

As shown in Figure 7, after clock synchronization, the uplink and downlink basically fluctuate on and off the 20 ms horizontal line, consistent with the results of the above delay distribution verification. The experimental results show that the synchronization on the actual experimental platform is consistent with the theoretical results, and the theoretically estimated clock phase offset and clock frequency offset are correct. Experiments show that the synchronous sensing effect can be achieved through the above clock delay compensation.

**FIGURE 8:**
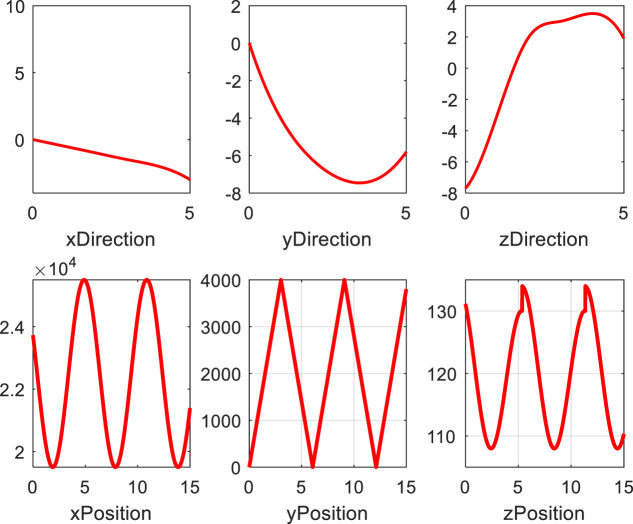
End position and angle of the bio-inspired mobile manipulator under synchronous sensing.

When the random delay distribution of sensor networks obeys the normal distribution, the mean square error of clock phase offset and clock frequency offset gradually reaches the lower bound with the increase of the number of observations. The experimental results show that the performance of estimating clock phase offset and clock frequency offset is better. Using synchronous sensing, the position and angle information of the end of the bio-inspired mobile manipulator is shown in Figure 8. As shown in Figure 8, in the synchronous sensing system, the angle and position information of the bio-inspired mobile manipulator can be fed back to the controller in time without time delay.

### 4.2 The Accuracy Verification of the Algorithm

In order to verify the correctness and effectiveness of the dynamic sliding mode controller designed above, we have carried out simulation experiments on the adaptive dynamic sliding mode controller designed above. The control object used in the simulation experiment is the nonholonomic bio-inspired mobile manipulator system shown in Figure 1. In the simulation, it is assumed that the desired trajectory of the bio-inspired mobile manipulator system is $z_{d}=\left[2\sin t,-3\cos t,\sin2t,\cos2t\right]$ , so that the centroid position of the mobile platform tracks the elliptical motion and the angular displacement of the two connecting rods tracks the sinusoidal motion. The parameters of the system are shown in Table 1.

**TABLE 1:** Key parameters of mobile platform and connecting rod.

Mass of mobile platform	$m_{0}$	50	Mass of connecting rod 1	$m_{1}$	4
Mass of connecting rod 2	$m_{2}$	3.5	Wheels distance	r	0.15
Lengths of connecting rod 1	$l_{1}$	0.6	Lengths of connecting rod 2	$l_{2}$	0.3
Moment inertia of platform	$J_{0}$	1.572	Moment inertia of rod 1	$J_{1}$	0.04
Moment inertia of rod 2	$J_{2}$	0.035	distance from the hinge point	$r_{1}$	0.3

The above controller parameters are selected to obtain $\theta_{LD},\theta_{RD},\theta_{1},\theta_{2}$ , as shown in Figure 8.

**FIGURE 9:**
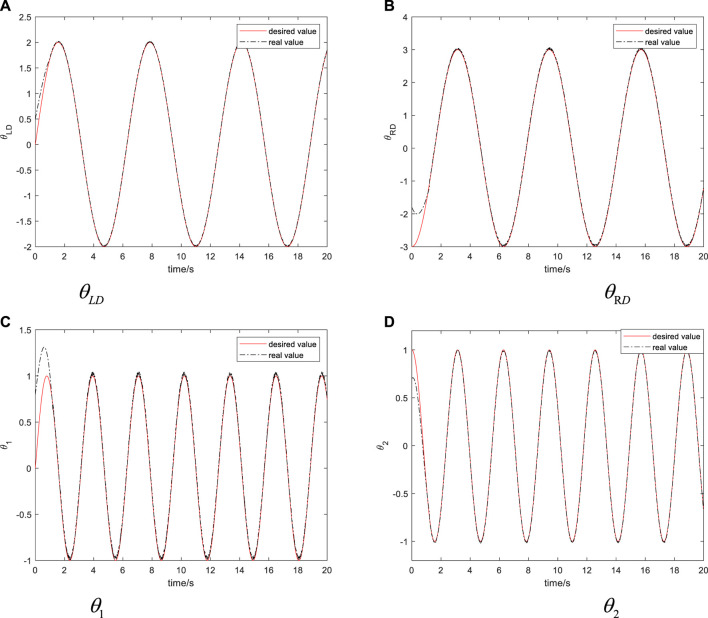
The simulation results of the bio-inspired mobile manipulator.

As can be seen from Figure 9 when considering the nonholonomic constraints of the bio-inspired mobile manipulator and the coupling effects of the mobile platform and the manipulator. Even if the initial position error of the system is large, each state in the system can quickly track the upper reference trajectory, which shows that the method proposed in this article can meet the requirements of the rapidity of the controller. As can be seen from Figure 9, the method proposed in this article can make the system reach the desired position quickly, the adjustment time is shorter, and the steady-state tracking error of the system is smaller. Hence, the control effect of the method proposed in this article is better. The simulation results show that the dynamic sliding mode controller designed by us successfully realizes the tracking of the given trajectory of the wheeled bio-inspired mobile manipulator system. Even in the case of large initial error and random disturbance, the tracking effect is also very good, and there is no chattering of system control.

### 4.3 The Verification of the Superiority of the Algorithm

**FIGURE 10:**
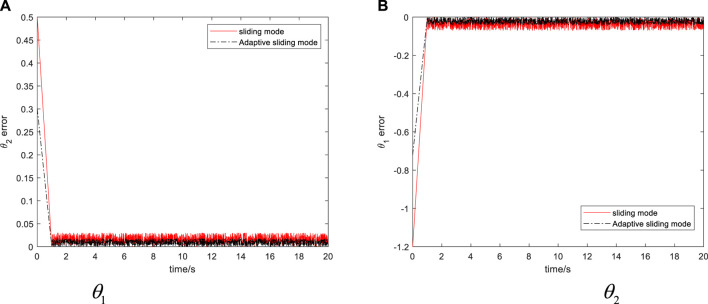
The simulation results of the bio-inspired mobile manipulator.

In order to compare with the traditional sliding mode control, the expected trajectory and initial state given by the two methods are the same, and the tracking error curve of the system is obtained, as shown in Figure 10.

Through the simulation example shown in Figure 10, it can be seen that the controller proposed in this article can not only ensure the stability of the system and the boundedness of tracking error but also ensure good tracking effect when the system dynamic model is uncertain or there is bounded disturbance in the system. Compared with the traditional sliding mode control, the controller proposed needs less system information and can realize the real-time estimation of unknown parameters so that the control system has the ability of anti-interference to the changes of parameters. All these show that the adaptive sliding mode control proposed in this article is correct and effective for the trajectory tracking problem of the bio-inspired mobile manipulator. After the experiment, the method proposed in this article can successfully complete the door opening experiment in a certain range. However, this method is mainly based on the coordinate measurement of synchronous sensor network and target recognition. Therefore, it is highly dependent on the measurement accuracy of the sensor itself, and the measurement error of the sensor itself will affect the final experimental results.

## 5 Conclusion

In this article, the adaptive control of the bio-inspired mobile manipulator based on synchronous sensing is studied. In order to realize synchronous sensing, this article focuses on the random delay distribution and the clock synchronization of adjacent nodes in wireless sensor networks. Firstly, the theoretical model of delay distribution is established according to the delay component, and it is verified that the random delay distribution of adjacent nodes obeys the normal distribution. Secondly, the maximum likelihood estimation method is used to calculate the clock phase offset and clock frequency offset under the theoretical model of adjacent nodes. The clock phase offset and frequency offset are compensated on the hardware platform to realize the clock synchronization of adjacent sensor nodes. This article studies the trajectory tracking control of bio-inspired mobile manipulator. Aiming at the uncertainty of model parameters and the boundedness of external disturbances, an adaptive sliding mode controller is proposed. In addition, based on the dynamic equation of the bio-inspired mobile manipulator, the sliding mode function about the tracking error is defined, and the torque controller is designed according to the defined sliding mode surface. In the design of a traditional sliding mode controller, the upper bound information of parameters must be known. However, in the actual system, the upper bound of parameters is often unknown. Therefore, an adaptive method is proposed to estimate the parameters online. The results show that the proposed algorithm has good results.

## Data Availability

The original contributions presented in the study are included in the article/Supplementary Material. Further inquiries can be directed to the corresponding author.

## References

[B1] Ahmad T. Abbas A. M. (2020). EEAC: An Energy Efficient Adaptive Cluster Based Target Tracking in Wireless Sensor Networks. J. Interdiscip. Maths. 23 (2), 379–392. 10.1080/09720502.2020.1731951

[B2] Almesaeed R. Al-Nasser A. Al-Junaid H. (2021). A Comprehensive Survey on Routing and Security in Mobile Wireless Sensor Networks. Int. J. Electron. Telecommunications 67 (3), 483–496. 10.24425/ijet.2021.137838

[B3] Asfour T. Paus F. Waechter M. Kaul L. Rader S. Weiner P. (2019). ARMAR-6: A High-Performance Humanoid for Human-Robot Collaboration in Real-World Scenarios. IEEE Robot. Automat. Mag. 26 (4), 108–121. 10.1109/MRA.2019.2941246

[B4] Bostelman R. Foufou S. Hong T. Shah M. (2018). Model of Mobile Manipulator Performance Measurement Using Sysml. J. Intell. Robot Syst. 92 (1), 65–83. 10.1007/s10846-017-0705-4 PMC6512825 31092980

[B5] Brahmi B. Driscoll M. El Bojairami I. K. Saad M. Brahmi A. (2021). Novel Adaptive Impedance Control for Exoskeleton Robot for Rehabilitation Using a Nonlinear Time-Delay Disturbance Observer. ISA Trans. 108, 381–392. 10.1016/j.isatra.2020.08.036 32888727

[B6] Chen T. Peng L. Yang J. Cong G. (2021a). Analysis of User Needs on Downloading Behavior of English Vocabulary APPs Based on Data Mining for Online Comments. Mathematics 9 (12), 1341. 10.3390/math9121341

[B7] Chen T. Yin X. Peng L. Rong J. Yang J. Cong G. (2021b). Monitoring and Recognizing Enterprise Public Opinion from High-Risk Users Based on User Portrait and Random Forest Algorithm. Axioms 10 (2), 106. 10.3390/axioms10020106

[B8] de Gea Fernández J. Mronga D. Günther M. Knobloch T. Wirkus M. Schröer M. (2017). Multimodal Sensor-Based Whole-Body Control for Human-Robot Collaboration in Industrial Settings. Robotics Autonomous Syst. 94, 102–119. 10.1016/j.robot.2017.04.007

[B9] Ding R. Zhang J. Xu B. Cheng M. Pan M. (2019). Energy Efficiency Improvement of Heavy-Load mobile Hydraulic Manipulator with Electronically Tunable Operating Modes. Energ. Convers. Manage. 188, 447–461. 10.1016/j.enconman.2019.03.023

[B10] Goswami R. Das S. Seal N. Pathak B. Neogi S. (2021). High-Performance Water Harvester Framework for Triphasic and Synchronous Detection of Assorted Organotoxins with Site-Memory-Reliant Security Encryption via pH-Triggered Fluoroswitching. ACS Appl. Mater. Inter. 13 (29), 34012–34026. 10.1021/acsami.1c05088 34255471

[B11] Habibzadeh H. Soyata T. Kantarci B. Boukerche A. Kaptan C. (2018). Sensing, Communication and Security Planes: A New challenge for a Smart City System Design. Computer Networks 144, 163–200. 10.1016/j.comnet.2018.08.001

[B12] Hao Z. Wang Z. Bai D. Tao B. Tong X. Chen B. (2021). Intelligent Detection of Steel Defects Based on Improved Split Attention Networks. Front. Bioeng. Biotechnol. 10.3389/fbioe.2021.810876 PMC8793735 35096796

[B13] Jiang D. Li G. Sun Y. Hu J. Yun J. Liu Y. (2021a). Manipulator Grabbing Position Detection with Information Fusion of Color Image and Depth Image Using Deep Learning. J. Ambient Intell. Hum. Comput 12 (12), 10809–10822. 10.1007/s12652-020-02843-w

[B14] Jiang D. Li G. Tan C. Huang L. Sun Y. Kong J. (2021b). Semantic Segmentation for Multiscale Target Based on Object Recognition Using the Improved Faster-RCNN Model. Future Generation Comput. Syst. 123, 94–104. 10.1016/j.future.2021.04.019

[B15] Jiang J.-J. Bu L.-R. Wang X.-Q. Li C.-Y. Sun Z.-B. Yan H. (2018). Clicks Classification of Sperm Whale and Long-Finned Pilot Whale Based on Continuous Wavelet Transform and Artificial Neural Network. Appl. Acoust. 141, 26–34. 10.1016/j.apacoust.2018.06.014

[B16] Liu C. Gao J. Bi Y. Shi X. Tian D. (2020). A Multitasking-Oriented Robot Arm Motion Planning Scheme Based on Deep Reinforcement Learning and Twin Synchro-Control. Sensors 20 (12), 3515. 10.3390/s20123515 PMC7349783 32575907

[B17] Liu X. Jiang D. Tao B. Jiang G. Sun Y. Kong J. (2021a). Genetic Algorithm-Based Trajectory Optimization for Digital Twin Robots. Front. Bioeng. Biotechnol. 1433, 793782. 10.3389/fbioe.2021.793782 PMC8784515 35083202

[B18] Liu Y. Jiang D. Yun J. Sun Y. Li C. Jiang G. (2021b). Self-Tuning Control of Manipulator Positioning Based on Fuzzy PID and PSO Algorithm. Front. Bioeng. Biotechnol. 1443, 817723. 10.3389/fbioe.2021.817723 PMC8873531 35223822

[B19] Lu W. Wu D. Lin J. Lu K. Wang D. Ye M. (2020). Initial Position Detection for Selective Compliance Assembly Robot Arm Manipulator Joint Based on an Improved High-Frequency Injection Method. Proc. Inst. Mech. Eng. J. Syst. Control. Eng. 234 (8), 912–921. 10.1177/0959651819892406

[B20] Lundeen K. M. Kamat V. R. Menassa C. C. McGee W. (2019). Autonomous Motion Planning and Task Execution in Geometrically Adaptive Robotized Construction Work. Automation in Construction 100, 24–45. 10.1016/j.autcon.2018.12.020

[B21] Mo Y. Jiang Z. Li H. Yang H. Huang Q. (2020). A Kind of Biomimetic Control Method to Anthropomorphize a Redundant Manipulator for Complex Tasks. Sci. China Technol. Sci. 63 (1), 14–24. 10.1007/s11431-019-9542-5

[B22] Nakanishi J. Itadera S. Aoyama T. Hasegawa Y. (2020). Towards the Development of an Intuitive Teleoperation System for Human Support Robot Using a VR Device. Adv. Robotics 34 (19), 1239–1253. 10.1080/01691864.2020.1813623

[B23] Outón J. L. Villaverde I. Herrero H. Esnaola U. Sierra B. (2019). Innovative Mobile Manipulator Solution for Modern Flexible Manufacturing Processes. Sensors 19 (24), 5414. 10.3390/s19245414 PMC6960724 31835307

[B24] Prianto E. Kim M. Park J.-H. Bae J.-H. Kim J.-S. (2020). Path Planning for Multi-Arm Manipulators Using Deep Reinforcement Learning: Soft Actor-Critic with Hindsight Experience Replay. Sensors 20 (20), 5911. 10.3390/s20205911 PMC7590214 33086774

[B25] Sun S. Zhao J. Tian X. Zhang J. (2019). Path Planning for Multiple Mobile Anchor Nodes Assisted Localization in Wireless Sensor Networks. Measurement 141, 124–136. 10.1016/j.measurement.2019.03.016

[B26] Tolosana R. Vera-Rodriguez R. Fierrez J. Morales A. Ortega-Garcia J. (2020). Deepfakes and Beyond: A Survey of Face Manipulation and Fake Detection. Inf. Fusion 64, 131–148. 10.1016/j.inffus.2020.06.014

[B27] Wu H. Guan Y. Rojas J. (2019). A Latent State-Based Multimodal Execution Monitor with Anomaly Detection and Classification for Robot Introspection. Appl. Sci. 9 (6), 1072. 10.3390/app9061072

[B28] Wu Y. Zhao F. Tao T. Ajoudani A. (2020). A Framework for Autonomous Impedance Regulation of Robots Based on Imitation Learning and Optimal Control. IEEE Robot. Autom. Lett. 6 (1), 127–134. 10.1109/LRA.2020.3033260

[B29] Xia K. Gao H. Ding L. Liu G. Deng Z. Liu Z. (2018). Trajectory Tracking Control of Wheeled Mobile Manipulator Based on Fuzzy Neural Network and Extended Kalman Filtering. Neural Comput. Applic 30 (2), 447–462. 10.1007/s00521-016-2643-7

[B30] Xiao F. Li G. Jiang D. Xie Y. Yun J. Liu Y. (2021). An Effective and Unified Method to Derive the Inverse Kinematics Formulas of General Six-DOF Manipulator with Simple Geometry. Mechanism Machine Theor. 159, 104265. 10.1016/j.mechmachtheory.2021.104265

[B31] Xu G.-H. Qi F. Lai Q. Iu H. H.-C. (2020). Fixed Time Synchronization Control for Bilateral Teleoperation mobile Manipulator with Nonholonomic Constraint and Time Delay. IEEE Trans. Circuits Syst. 67 (12), 3452–3456. 10.1109/TCSII.2020.2990698

[B32] Yang Z. Jiang D. Sun Y. Tao B. Tong X. Jiang G. (2021). Dynamic Gesture Recognition Using Surface EMG Signals Based on Multi-Stream Residual Network. Front. Bioeng. Biotechnol. 9, 779353. 10.3389/fbioe.2021.779353 34746114 PMC8569623

[B33] Zhao G. Jiang D. Liu X. Tong X. Sun Y. Tao B. (2021). A Tandem Robotic Arm Inverse Kinematic Solution Based on an Improved Particle Swarm Algorithm. Front. Bioeng. Biotechnol. 10.3389/fbioe.2021.832829 PMC9162496 35662837

